# Thoracic ultrasound use in hospitalized and ambulatory adult patients: a quantitative picture

**DOI:** 10.1186/s13089-024-00359-4

**Published:** 2024-02-21

**Authors:** N. Xirouchaki, M. Bolaki, C. Psarologakis, E. Pediaditis, A. Proklou, E. Papadakis, E. Kondili, D. Georgopoulos

**Affiliations:** Intensive Care Medicine Department, University Hospital of Heraklion, University of Crete, Voutes, 71110 Heraklion, Crete Greece

**Keywords:** Thoracic ultrasound, Ambulatory, Malignancy, Pregnancy, Diaphragmatic dysfunction

## Abstract

**Introduction and objectives:**

Thoracic ultrasound (TUS) has been established as a powerful diagnostic and monitoring tool in the Intensive Care Unit (ICU). However, studies outside the critical care setting are scarce. The aim of this study was to investigate the value of TUS for hospitalized or ambulatory community patients.

**Materials and methods:**

This was a retrospective study conducted from 2016 to 2020 in the TUS clinic at Heraklion University Hospital. TUS examination was performed using a standard ultrasound machine (EUB HITACHI 8500), and a high-frequency microconvex probe (5–8 MHz). Patients had been referred by their primary physician to address a range of different questions. The various respiratory system entities were characterised according to internationally established criteria.

**Results:**

762 TUS studies were performed on 526 patients due to underlying malignancy (*n* = 376), unexplained symptoms/signs (*n* = 53), pregnancy related issues (*n* = 42), evaluation of abnormal findings in X-ray (*n* = 165), recent surgery/trauma (*n* = 23), recent onset respiratory failure (*n* = 12), acute respiratory infection (*n* = 66) and underlying non-malignant disease (*n* = 25). Pleural effusion was the commonest pathologic entity (*n* = 610), followed by consolidation (*n* = 269), diaphragmatic dysfunction/paradox (*n* = 174) and interstitial syndrome (*n* = 53). Discrepancies between chest X-ray and ultrasonographic findings were demonstrated in 96 cases. The TUS findings guided invasive therapeutic management in 448 cases and non-invasive management in 43 cases, while follow-up monitoring was decided in 271 cases.

**Conclusions:**

This study showed that TUS can identify the most common respiratory pathologic entities encountered in hospitalized and community ambulatory patients, and is especially useful in guiding the decision making process in a diverse group of patients.

**Supplementary Information:**

The online version contains supplementary material available at 10.1186/s13089-024-00359-4.

## Introduction

Thoracic ultrasound (TUS) has emerged in recent years as a powerful, easily repeatable bedside diagnostic and monitoring tool. Based on its ability to identify a wide variety of respiratory system diseases, TUS has an established role in critically ill patients and plays a pivotal effect on clinical decision making [1, 2]. Indeed, a consensus of experts has introduced TUS as one of the required elements in achieving competence in general critical care ultrasound [3–5]. However, despite its extended use in ICUs over the last decade, little is known about the value of TUS in non-critically ill hospitalized or ambulatory community patients. Outside of critical care settings, TUS has mainly been applied in heart failure patients to assess and monitor pulmonary congestion, and had an established role during the COVID 19 pandemic [6, 7]. Surprisingly, the value of this powerful tool has yet to be systematically studied, and no sufficient data are available on its application in non-critically ill hospitalized patients or community patients with various pathologic conditions.

Following our extensive long-term experience with TUS in critically ill patients in our hospital, a TUS clinic for non-critically ill patients was established. The aim of this study was to focus on: (1) analyzing advances in the knowledge of TUS application and the related main protocols adopted; (2) discussing how and when thoracic ultrasound should be used on hospitalized non-critically ill patients, as well as on community ambulatory patients; (3) demonstrating the possible future development of TUS in that context.

## Methods

This retrospective study was conducted from November 2016 to May 2020. Approval for the anonymous use of data was obtained from the Hospital’s Ethics Committee. Patients were referred to the TUS clinic by their primary physicians to address questions related to: (1) underlying malignancy (Q1), (2) unexplained symptoms/signs (Q2), (3) context of pregnancy (Q3), (4) further evaluation of chest X-ray (Q4), (5) recent thoracic surgery/trauma (Q5), (6) unexplained recent onset respiratory failure (Q6), (7) respiratory infections (Q7) and (8) underlying non-malignant disease with possible respiratory system involvement (Q8). Patients were included in the study providing that they fulfilled one of the criteria mentioned above, they agreed to participate in the study, there was complete information on their health record and they could be transferred to the TUS clinic for TUS examination. Those refusing to take part in the study were excluded.

TUS examination was performed using a standard ultrasound machine (EUB HITACHI 8500), and a high frequency microconvex probe (5–8 MHz). TUS examination was performed in a fully equipped echography Lab with nursing support. A 12-region protocol was used, with patients in sitting and/or lying positions. All TUS examinations were performed or closely supervised by an expert operator (NX). Ethics committee approval and informed consent were obtained.

The various respiratory system pathologies were characterized using well defined ultrasonographic signs (Additional file 1: Table S1) [8–20].

## Results

### General

Seven hundred and sixty-two TUS studies were performed on 526 patients from November 2016 to May 2020. Patient characteristics, the number of TUS studies per patient and the referral Department are shown in Table 1 (Additional file 1: Fig S1a).

**Table 1:** Baseline characteristics

Characteristics	
Number of patients^*^	526^a^
Number of lung ultrasound studies	762^a^
Age	60 (48–70)^b^
Male/female	295/206^c^
No thoracic ultrasound study/pt	
1	412^a^
2	65^a^
3	20^a^
4	16^a^
5	4^a^
6	3^a^
7	1^a^
8	2^a^
9	1^a^
10	1^a^
16	1^a^
Origin	
Hospitalized	648
Ambulatory	114
Department of referral	
Pulmonary Department	196^a^
Oncology Department	314^a^
Hematology Department	40^a^
Obstetrics Department	16^a^
Outpatient	114^a^
General Surgery Department	34^a^
Other departments^$^	48^a^

^a^values are expressed as absolute numbers

^b^values are expressed as median (IQR)

^c^the gender in 25 out of 526 pts is not known

^$^Thoracic Surgery, Neurosurgery, Gastroenterology, Rheumatology, Internal Medicine, ICU^#^, ED^^^, Cardiology

^#^*ICU* intensive care unit

^^^*ED* Emergency Department

**Fig. 1:**
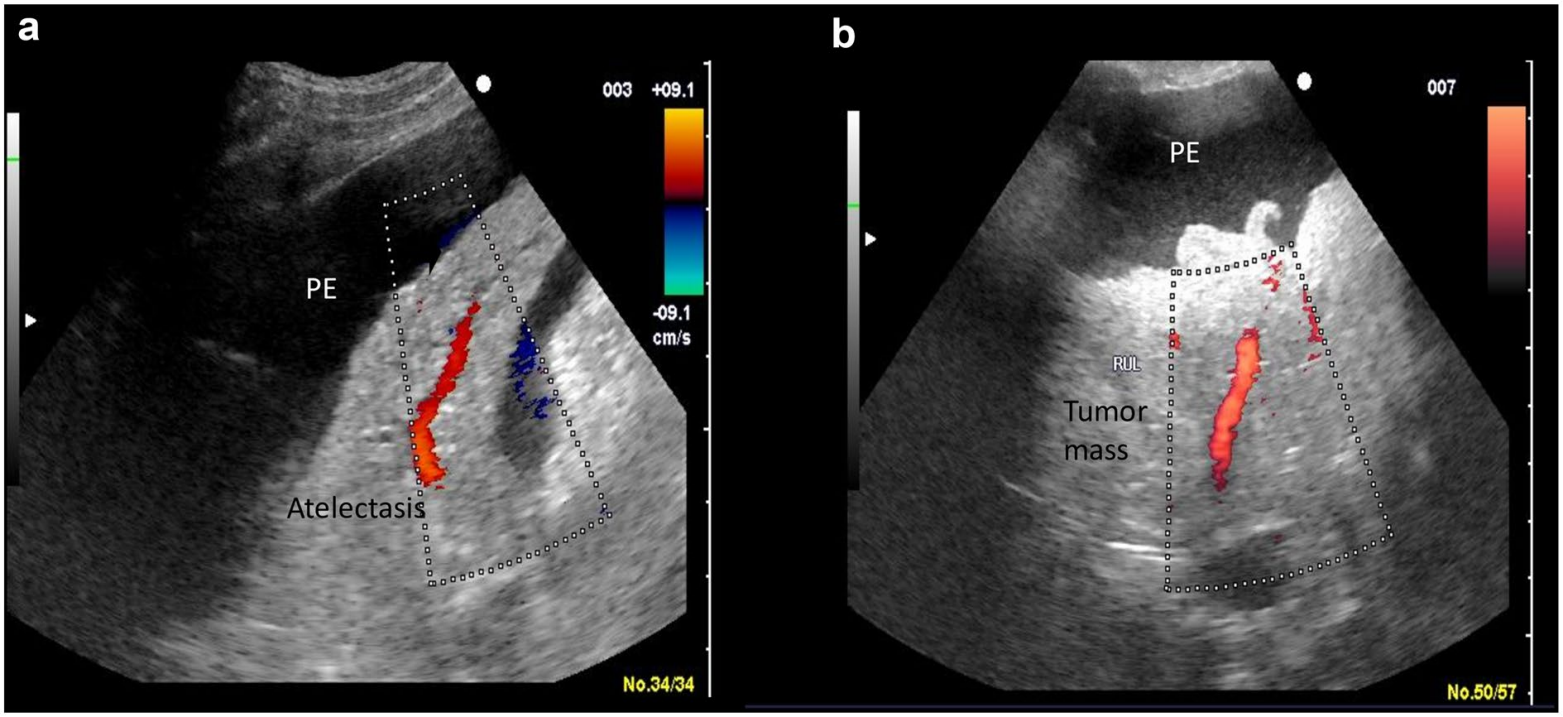
**a** Compressive atelectasis associated with large pleural effusion (PE). Colour doppler was used to depict the vessel inside the consolidated lung. **b** Right upper lobe tumor. Notice the augmented vascularization, the irregular boundaries and the complete loss of aeration. *PE* pleural effusion

**Fig. 2:**
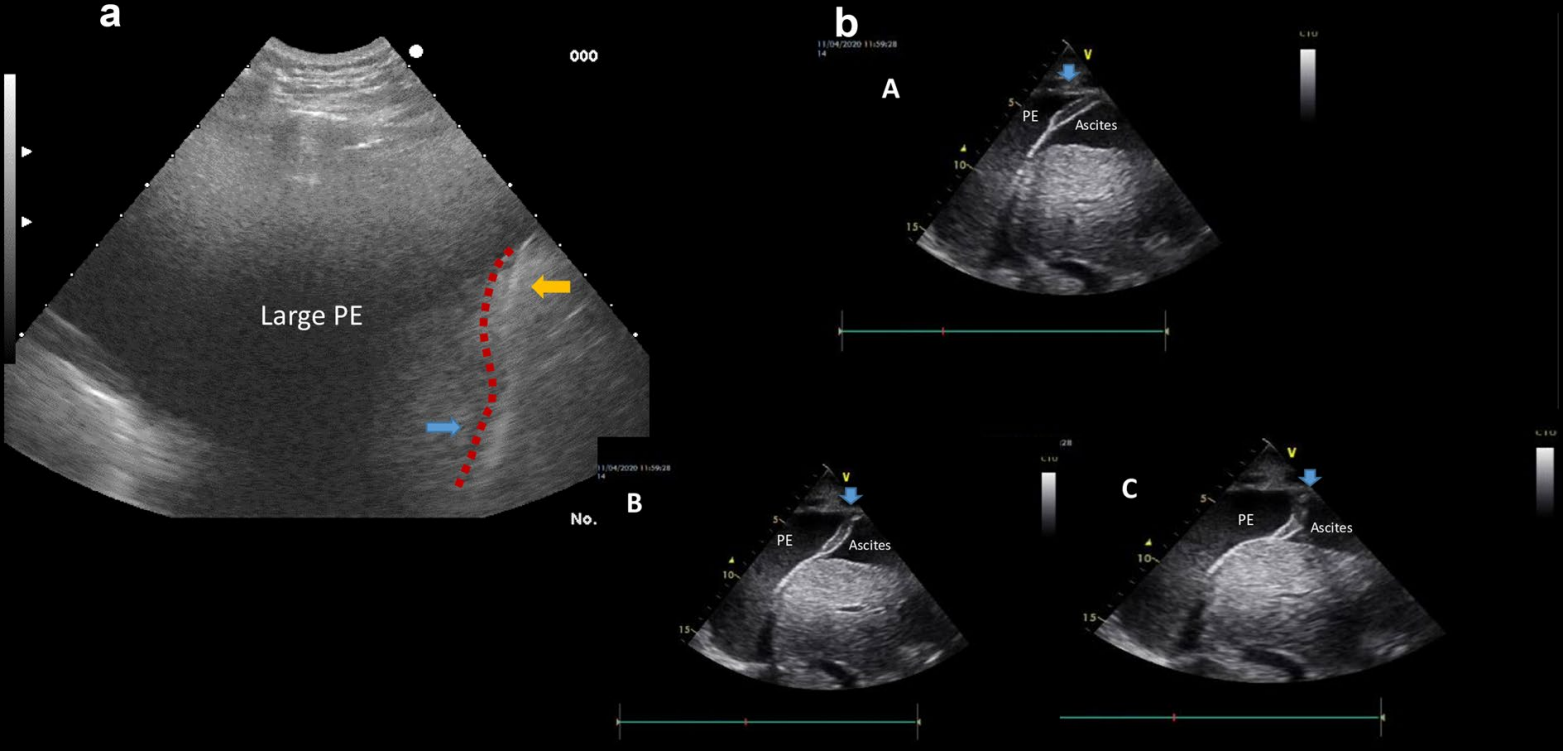
**a** Diaphragm dysfunction with atypical or paradoxical movement due to large pleural effusion (PE). Red dot line delineates the abnormally shaped diaphragm. Yellow and blue arrows indicate the reverse movement during inspiration. Note that when this sign is present, pleural fluid should be evacuated immediately. **b** Large pleural effusion (PE) and ascites. The blue arrow indicates the three-layer structure of the diaphragm floating in the fluid: **a** during expiration; **b** during inspiration, with abnormal shape; and **c** during deep inspiration, with the abnormal shape indicating paradoxical movement

Underlying malignancy (Q1) and the further evaluation of abnormal X-ray findings (Q4) were the two most common reasons for performing TUS, accounting for 49% and 21.6% of studies, respectively (Table 2, Additional file 1: Fig S1b). Only 57 out of 762 studies (7.5%) were characterized as normal (Table 2). Pleural effusion was the commonest pathologic entity, followed by consolidation, diaphragmatic dysfunction or paralysis and interstitial syndrome (Additional file 1: Tables S2 and S3) (Additional files 1, 3: Figs. S1c. and S3b). Ultrasonographic characteristics of loculation were observed in 120 out of 610 cases of pleural effusion (Additional file 1: Table S2) (Additional file 3: Fig. S3a). Consolidation cases were mostly due to atelectasis (*n* = 225) (Fig. 1a), while pneumonia was diagnosed in 44 cases. In 11 cases, there were findings consistent with pulmonary embolism; in all instances, this diagnosis was confirmed by spiral CT performed immediately after TUS (Table 2). Signs of diaphragmatic dysfunction/paradox or paralysis were mainly due to malignancy (*n* = 175). (Fig. 2a, b).

**Table 2:** TUS results

Number of patients	526^a^
Number of thoracic ultrasound studies	762^a^
Question for TUS referral	
Q1: underlying malignancy	376^b^
Q2: unexplained symptoms/signs	53^b^
Q3: pregnancy related	42^b^
Q4: further evaluation of abnormal X-ray findings	165^b^
Q5: recent surgery/trauma	23^b^
Q6: recent onset respiratory failure	12^b^
Q7: respiratory infections	66^b^
Q8: underlying non-malignant disease with possible respiratory system involvement	25^b^
Thoracic ultrasound findings^c^	
Pleural effusion	610^a^
Consolidation^d^	269^a^
* Atelectasis*	225^a^
* Pneumonia*	44^a^
Pulmonary embolism	11^a^
Interstitial syndrome	53^a^
Diaphragm dysfunction (malignant/non malignant)	168/6^a^
Diaphragm paralysis	7
Pneumothorax	5^a^
Normal	57^a^
Discrepancies^e^	
Yes	96^a^
No/don’t know	666^a^
Decision	
Invasive (thoracentesis, drainage, chest tube, surgery)	448^a^
Non-invasive (antibiotics, diuresis, other examinations order)	43^a^
Monitoring/Follow up/No intervention	271^a^

^a^Values are expressed as absolute numbers

^b^Number of lung ultrasound examinations per question (total *N* = 759), values are expressed as absolute numbers

^c^More than one lung ultrasound finding per study is possible

^d^Consolidation can be subdivided into atelectasis and pneumonia

^e^Discrepancies between chest X-ray or CT finding and ultrasonographic findings

Six cases of diaphragmatic dysfunction were observed during investigation of underlying neuromuscular disease or recent onset of hypercapnic respiratory failure. Findings compatible with pneumothorax were observed in 5 cases (2 secondary, 1 primary, 2 iatrogenic) (Additional file 2: Fig. S2a).

Discrepancies between chest X-ray and ultrasonographic findings existed in 96 cases (Table 2 and Additional file 1: Table S4). In most cases, chest X-ray or CT revealed significant pleural effusion, while TUS showed no or small pleural effusion (*n* = 67). In 6 cases TUS demonstrated pleural effusion not depicted in chest X-ray, in 7 cases the pathology (e.g., infiltrate, nodule, mass) depicted in chest X-ray was not confirmed by TUS, and in 16 cases a new pathology (e.g., consolidation, pneumothorax, abscess) was revealed by TUS not shown in chest X-ray or CT.

Overall, management was changed directly as a result of information provided by the lung ultrasound operator in 491 cases. In 448 cases the change in a patient’s management involved invasive interventions (thoracentesis, drainage, chest tube insertion or removal, surgery, bronchoscopy and CT-guided biopsy) and in 43 cases non-invasive interventions (initiation or change of antibiotics, diuretics, referral for further imaging tests, hospital admission) (Additional file 2: Fig. S2b). In several cases (*n* = 271), follow-up without intervention was decided on.

#### Subgroup analysis

##### Pregnancy

Forty-two TUS studies were performed in the context of pregnancy (26 pregnant, 7 post-partum, 9 women with ovarian hyperstimulation syndrome) (Additional file 1: Table S5). The main reasons for examination were respiratory symptoms (*n* = 5), pleural effusion evaluation and management (*n* = 29) or monitoring of a lung abnormality (*n* = 8). Based on the ultrasound features, monitoring (26 cases), thoracentesis (10 cases), surgery referral (2 cases) was decided, whereas 4 cases revealed normal findings without need for further examination. In case of pregnancy the elevation of diaphragm > 2 cm was a common finding.

##### Cancer patients

376 cancer patients underwent TUS examination for new symptoms (*n* = 252), evaluation of pleural effusion (*n* = 136), changes in chest x ray (*n* = 24) and monitoring in 21 patients (Additional file 1: Table S5). TUS revealed pleural effusion (*n* = 293), obstructive or compressive atelectasis (*n* = 233), loculated effusion/pleural thickening or empyema (*n* = 56) (Fig.S3a), pneumonia (*n* = 10), cancer infiltration or tumour mass (*n* = 10) (Fig. 1b), haemothorax, chylothorax and pneumothorax (*n* = 4, 2 and 1, respectively). In view of the specific ultrasound findings, special attention was paid to segmental or total diaphragm paradox in 168 patients, and diaphragm paralysis in 7 cases. (Fig. 2b). Each TUS examination could have shown more than one pathologic entity. According to these findings, 336 invasive interventions were performed as follows: pleural effusion drainage in 186 patients, diagnostic thoracentesis in 111 patients, chest tube or flexima placement/removal in 39 cases. Non-invasive interventions included monitoring in 25 cases, surgery suggestion or diuresis in 6 patients. A diverse diagnosis was made in 55 patients, causing a change in clinical decision (Additional file 1: Table S6).

## Discussion

The main finding of this study is that in a large group of hospitalized and community ambulatory patients, TUS identifies a range of pathologic entities and changes management interventions. There are many studies validating application of the technique for community-acquired and ventilator-associated pneumonia, pneumothorax, and acute respiratory failure. However, in this study TUS was applied in diverse patient populations, including patients with underlying malignancy, pregnancy and trauma/surgical patients [21–23].

Lung ultrasound represents an emerging and useful imaging technique in the diagnosis and management of pulmonary and pleural diseases. Thanks to its simplicity and non-invasive nature, it can be useful as a bedside tool for establishing diagnosis, monitoring and guiding management strategies in various clinical setting [7]. It can definitely serve as a basic application in critically ill patients, as it demonstrates sensitivity and specificity ranging from 90% to 100% (using CT as the “gold standard”) in the assessment of different respiratory disorders encountered in the ICU [2, 20, 24]. We have previously shown that lung ultrasound has better diagnostic performance than CXR for common pathologic entities such as consolidation, interstitial syndrome, pneumothorax and pleural effusion [24]. In addition, it has a significant impact on clinical decision making in critically ill patients [25]. However, outside the critical care setting data are not so powerful. The limitations associated with lung ultrasound need to be mentioned, irrespective of the fact that they do not outweigh its benefits. First, LU is a highly operator-dependent imaging modality, which requires training. Subcutaneous emphysema and thoracic dressings, conditions common in the critical care setting, can prevent image acquisition and make the examination impossible from a technical point of view. Moreover, ultrasound can underestimate lesions that involve deep lung layers [1].

Although TUS can guide diagnosis in a variety of chest diseases in the emergency department [26] and affect therapeutic management [27], its use is not widespread among clinicians. This is despite the existing literature supporting its usefulness: studies have shown that lung ultrasound can be used in the diagnosis and follow up of community-acquired pneumonia [28–30], while TUS has been found to be a significant diagnostic tool in evaluating and managing patients with pleural effusions [31]. It has also been shown to be a valuable tool in the assessment and monitoring of lung congestion in cardiogenic pulmonary edema [32–34] or in guiding diuretic treatment in patients with heart failure [35]. Recently, TUS has been used in the evaluation of connective tissue disease-associated interstitial lung disease [36, 37]. The assessment of diaphragm thickness, thickening fraction, and excursion in both ambulatory and mechanically ventilated patients could be crucial to identifying diseases responsible for diaphragm dysfunction [38]. Serial diaphragmatic assessment by ultrasound may lead to earlier non-invasive ventilation initiation in patients with amyotrophic lateral sclerosis [39]. During the COVID-19 pandemic, lung ultrasound was recognized as an invaluable tool in the diagnosis and monitoring of the disease in the lung, greatly influencing the clinical-decision making process in affected patients [40, 41]. Obviously, the higher sensitivity of lung ultrasound compared to chest radiography is an advantage, though it can also prompt diagnoses of conditions that erroneously guide unnecessary treatment modalities. Integrated clinical and imaging skills are needed to achieve appropriate patient management in this setting [42].

In this study, the vast majority of TUS examinations were performed due to underlying malignancy. Primary or secondary lung cancer can present with pleural effusion, pulmonary edema, post-obstructive pneumonia or ascites, which can be assessed and managed successfully by the clinician with point-of-care ultrasound [43]. In a cross-sectional study of 53 patients with confirmed bronchogenic carcinoma, thoracic ultrasound had a significant complementary role to computed tomography in the diagnosis and staging of this type of cancer, especially if peripherally located [18]. In a recent meta-analysis, although TUS was not useful as a ruling-out test for malignant pleural effusion, the identification of pleural nodularity could motivate further investigation if there is a strong suspicion it is present [44]. In this study, a large number of patients with malignancy were evaluated and TUS was used to guide invasive procedures. Primary or secondary malignant disease was associated with significant bilateral asymmetrical pleural effusions. The main finding of this study was the paradoxical movement of the diaphragm due to large malignant effusion. Indeed, almost in 58% (168/293) of examinations, diaphragmatic inversion or paradoxical movement of the hemi-diaphragm was detected during inspiration, and full recovery was observed after pleural fluid evacuation. Although diaphragmatic paradoxical movement has already been reported [13, 45], this study is the first to describe this reversible phenomenon in a considerable number of malignant effusions.

Additionally, we performed 42 TUS studies in the context of pregnancy (pregnancy, post-partum, ovarian hyperstimulation syndrome), thus avoiding exposure to radiation in this vulnerable group of patients. TUS patterns have already been described in healthy parturients [46], in women during labor [47] as well as in pregnant women with preeclampsia [48]. Lung ultrasound has been proved to be a valuable tool in identifying excess lung water in severe preeclamptic patients [49] and predicting interstitial syndrome and hemodynamic profile in this group [50].

The present study is the first to describe the ultrasonographic findings and clinical course in a diverse group of ambulatory patients presenting to the lung ultrasound department for a variety of reasons, e.g., due to an underlying clinical condition, or an unexplained clinical picture or imaging tests. The strengths of this study are the large number of participants, the application of TUS as a single diagnostic method in most cases, and the use of TUS as a decision making and monitoring tool. Limitations include the retrospective design of the study, single-center conduction, the inevitable existence of (limited) missing data, and the fact TUS findings were not confirmed by computed tomography, which is regarded as the gold standard technique. However, a previous study from the same center has proved the high diagnostic performance of TUS and its applicability as alternative to CT.

## Conclusion

Thoracic ultrasound is capable of identifying a variety of pathologic conditions affecting hospitalized and community ambulatory patients, and can be useful in guiding therapeutic interventions. This study implies that it is potentially a promising bedside tool in the clinician’s armamentarium, and something that future prospective studies are expected to further explore.

### Supplementary Information

**Additional file 1: Table S1.** Definition of various respiratory system pathologies. **Table S2.** Pleural effusion. **Table S3.** Ιnterstitial syndrome. **Table S4.** Discrepancies. **Table S5.** ΤUS results related to pregnancy. **Table S6.** TUS results related to malignancy. **Figure S1.** **a**, **b**, **c** Department of referral, question for TUS referral and thoracic ultrasound findings, respectively

**Additional file 2: Figure S2.** **a** Pneumothorax in a patient presented with hypoxemia after many attempts of diagnostic thoracentesis at the ward. Red arrow: Pleural line. Blue arrows: A lines. M-mode displays the stratosphere–barcode sign. Yellow arrow. **b** Echo-guided drainage of a large pleural effusion. The blue arrow indicate the needle linked with the acoustic shadow inside the pleural cavity. Notice the consolidated lung, the large pleural effusion (PE) and the diaphragm with well-defined borders and normal shape (white arrow).

**Additional file 3: Figures S3.** **a** TUS after talc pleurodesis. Multiple diaphragms and pockets in the pleural space. Notice the normal shape of the diaphragm indicating the absence of inflammatory process. **b** Left: B-mode clearly shows the three diaphragmatic layers. Black arrow indicates the inner diameter corresponding to the diaphragmatic thickness. Right: Points of estimation of diaphragmatic thickness during the respiratory cycle. Red arrow: maximal thickness during inspiration and white arrow during expiration.

## Data Availability

Not applicable.

## Abbreviations

TUS: Thoracic ultrasound
ICU: Intensive Care Unit
CXR: Chest X-ray
CT: Computed tomography
