# Supplementary material for: Thoracic ultrasound use in hospitalized and ambulatory adult patients: a quantitative picture
Source: Ultrasound J. 2024 Feb 21;16:11. doi: 10.1186/s13089-024-00359-4 (PMC10881936; doi:10.1186/s13089-024-00359-4)
Supplement: Supplementary file 1 — Additional file 1: Table S1. Definition of various respiratory system pathologies. Table S2. Pleural effusion. Table S3. Ιnterstitial syndrome. Table S4. Discrepancies. Table S5. ΤUS results related to pregnancy. Table S6. TUS results related to malignancy. Figure S1. a, b, c Department of referral, question for TUS referral and thoracic ultrasound findings, respectively [file 13089_2024_359_MOESM1_ESM.docx]

**Additional file**

**"Thoracic ultrasound use in hospitalized and ambulatory adult patients: a quantitative picture"**

N. Xirouchaki, M. Bolaki, Ch. Psarologakis, E. Pediaditis, A. Proklou, E. Papadakis, E. Kondili, D. Georgopoulos

Intensive Care Medicine Department, University Hospital of Heraklion, University of Crete, Heraklion, Crete, Greece

Address for correspondence: Nektaria Xirouchaki MD, phD

Intensive Care Unit,

Heraklion University Hospital

Voutes 71110, Heraklion

Crete- Greece

Email: nxirouchaki@gmail.com

Abbreviations: TUS: thoracic ultrasound

ICU: Intensive Care Unit

CXR: Chest X-ray

CT: Computed tomography

Keywords: thoracic ultrasound, ambulatory, malignancy, pregnancy, diaphragmatic dysfunction

Declarations of interest: none

This research did not receive any specific grant from funding agencies in the public,

commercial, or not-for-profit sectors

**Definition of various respiratory system pathologies**

1. **Pleura TUS**
2. Pleural effusion

Pleural effusion was determined as a hypoechoic, echoic or hyperechoic structure, containing isoechoic particles or septations in inflammatory pleural diseases [8]. Pleural effusion was divided in acute and chronic, the latter characterized by thick parietal pleura (>10mm), fluid colour sign, increased diaphragmatic thickness and ill-defined borders of the diaphragm [9, 10]. Fluid volume was estimated using the previous validated formula: volume (ml) = 20×Sep (mm), where Sep corresponds to maximal pleural separation at the lung base [10]. The pleural effusion according to the fluid volume was divided into small (20-150 ml), medium (150-500 ml) and large (>500 ml) [11]. Additional signs related to fluid volume were used, such as lobar or total atelectasis, changes in heart position and floating lung. Diaphragm deformation was used as an index of immediate pleural fluid evacuation.

Loculated effusion was characterized by the presence of fibrous strings and mobile or immobile septations with encapsulated liquid. In this case doppler or power doppler was used in differentiating lung tissue from infectious areas behind the pleura [12]. Haemothorax was characterized by large echogenic effusion containing floating thrombus that swirl with respiration or cardiac motion[13].

1. Pneumothorax

# Pneumothorax was diagnosed when complete abolition of the lung sliding was present. In M-mode the complete abolition of the lung sliding was expressed with barcode or stratosphere sign (parallel lines beneath the pleural line). The lung point specific sign for pneumothorax was examined (junction between sliding lung and absent sliding), and A line sign (only A lines visible) was used as well. The presence of the B lines excluded the diagnosis of pneumothorax [14].

1. Diaphragm

The examination of the diaphragm included inspection and displacement. Displacement >15mm was considered as normal. Diaphragmatic thickening fraction during inspiration (TF), an index of diaphragmatic contraction, was also examined[15-17]. TF is expressed as percentage and calculated as follows: TF= (TEI-TEE) *100/TEE, where TEI and TEE are diaphragmatic thickness at end inspiration and end expiration, respectively[17]. TF less than 25% is indicative of diaphragmatic dysfunction. Segmental or total hemi diaphragm paradox movement and deformation are also signs of diaphragmatic dysfunction. TF close to zero and/or cephalad movement during inspiration indicate diaphragmatic paralysis. In case of significant pleural effusion, the displacement and paradox movement was examined after fluid evacuation.

1. **Lung TUS**
2. Pneumonia

Pneumonia appeared as small consolidation with local B lines associated with subpleural consolidation. In some cases, lung hepatisation with lobar distribution was present. Consolidation due to pneumonia was also characterized by dynamic air bronchogram (strong echogenic structure with air moving through bronchi), linear or dendritic, visible till the peripheral bronchi. A tree-like distribution pattern of pulmonary vessels accompanying the bronchial tree in colour‐flow Doppler examination was an additional sonographic finding [12].

1. Atelectasis

Atelectasis was characterized by an iso-echoic tissue like structure associated by static air bronchogram (numerous scatter echogenic points created by entrapped air/infectious material within the consolidated region). Pleural effusion of moderate size was associated with total/lobar lung atelectasis. Compression atelectasis was associated with large pleural effusion and floating lung.

1. Tumor mass

Tumor was usually located nearby the thoracic cavity, surrounded by pleural fluid. Sonographic signs were the irregular morphology, the iso-echoic non-aerated structure, with irregular borders and the high vascularity within the mass (video fig.). In cases of extensive malignant disease diffuse coalescent B lines were found [18].

1. Pulmonary embolism

Pulmonary embolism was suspected when pleural based triangle or polygonal hypoechoic infracts located in a specific lung area for more than two pleural spaces were observed. The absence of other pathological ultrasound findings is in favour of the diagnosis of pulmonary embolism. [19].

1. Interstitial syndrome

Interstitial syndrome was defined by the presence of diffuse B lines in both lungs. B lines are well defined hyperechoic artifacts, arising from the pleural line and moving with the lung sliding when lung sliding is present. Multiple coalescent B-lines in all examined fields with bilateral pleural fluid and thin pleura were associated with the diagnosis of congestive heart failure [20]. B lines, 7 ± 1 mm apart, with thick nodular and interrupted pleura associated with subpleural lesions were associated with the diagnosis of pulmonary fibrosis [20]. Regarding B-lines, focal B-lines may be present in a normal lung, and a focal (localized) sonographic pattern of interstitial syndrome may be seen in various pathologic conditions [4].

The TUS operator presented the information to the primary physician and was not involved in the decision making process. Discrepancies between chest x –ray or CT and TUS findings were also reported.

**Table S1: Definition of various respiratory system pathologies**

| **PLEURA TUS** |  |  |  |
| --- | --- | --- | --- |
|  | 1. **PLEURAL EFFUSION** |  |  |
|  |  | ACUTE | Anechoic / Hypoechoic  Lateral or bilateral, various size |
|  |  | CHRONIC | Thick parietal pleura >10mm  Fluid colour sign  Echogenic  Ill-defined borders of the diaphragm |
|  |  | SMALL | 20-150ml |
|  |  | MEDIUM | 150-500ml |
|  |  | LARGE | >500 ml |
|  |  | LOCULATED | Echogenic, usually Lateral  Immobile Septations  Fibrous strings |
|  |  | HEMOTHORAX | Echogenic effusion  Moving particles within  Floating thrombus moving with respiration or cardiac motion |
|  |  | PLEURODESIS | Thin diaphragms and pockets all over the pleural space. |
|  |  | NEED IMMEDIATE EVACUATION | When diaphragm paradoxical movement is present |
|  |  |  |  |
|  | 1. **PNEUMOTHORAX** |  | Complete abolition of lung sliding |
|  |  |  | Lung point |
|  |  |  | A-line sign |
|  |  | EXCLUDE THE DIAGNOSIS | Presence of B lines |
|  |  |  |  |
|  | 1. **DIAPHRAGM** | DISPLACEMENT | Normal>15mm |
|  |  | FUNCTION TF% | Dysfunction<25% |
|  |  | PARALYSIS | Cephalad movement during inspiration |
|  |  | DEFORMATION  SEGMENTAL OR TOTAL WITH PARADOXICAL MOVEMENT | Excessive fluid accumulation in pleural cavity. |
| **LUNG TUS** |  |  |  |
|  | 1. **PNEUMONIA** |  | Local B-lines |
|  |  |  | Consolidation of various size |
|  |  |  | Lung hepatization frequently when Lobar distribution |
|  |  |  | Dynamic air bronchogram |
|  |  |  | Thick diaphragm with ill-defined borders |
|  |  |  | Echogenic effusion ipsilateral |
|  |  |  |  |
|  | 1. **ATELECTASIS** |  | Iso-echoic pattern with no aeration |
|  |  |  | Static air bronchogram |
|  |  |  | Normal diaphragm three layers structure |
|  |  |  | PE of Moderate size |
|  |  |  | Floating lung  Compressive atelectasis due to large effusion |
|  |  |  |  |
|  | 1. **TUMOR MASS** |  | Adjacent to thoracic cavity |
|  |  |  | Irregular morphology - borders |
|  |  |  | Iso-echoic non aerated area |
|  |  |  | High Vascularity |
|  |  |  |  |
|  | 1. **PULMONARY EMBOLISM** |  | Regional - Hypoechoic infracts triangle polygonal pleural based |
|  |  |  |  |
|  | 1. **INTERTITIAL SYNDROME** |  | Bilateral Diffuse B lines |
|  |  | ACUTE - CHF | Diffuse coalescent B-lines |
|  |  |  | Frequently Thin pleural line |
|  |  |  | Ipsilateral or bilateral small effusion |
|  |  | ACUTE – Infection - ALI | Diffuse coalescent B-lines |
|  |  |  | Slow sliding, Interrupted pleura |
|  |  |  | Subpleural consolidation |
|  |  |  |  |
|  |  | CHRONIC | Diffuse B7 lines >7mm |
|  |  |  | Thick nodular pleura |
|  |  |  | Subpleural lesions |
|  |  |  | Slow sliding, Lung aeration loss |

**Table S2: Pleural effusion**

| **Total number of TUS^#^ studies with pleural effusion finding** | 610 |
| --- | --- |
| **Size of pleural effusion** |  |
| Small | 320 |
| Medium | 90 |
| Large | 200 |
| **Loculated** | 120 |
| **Decision** |  |
| Diagnostic thoracentesis | 169 |
| Drainage | 198 |
| Chest tube/catheter or pleurodesis | 42 |
| Surgical | 24 |
| Conservative | 177 |
|  |  |

# TUS; thoracic ultrasound

**Table S3: Ιnterstitial syndrome**

| **Total number of TUS^#^ studies with interstitial syndrome finding** | 53 |
| --- | --- |
| Pulmonary oedema | 16 |
| Fibrosis | 16 |
| Cancer infiltrations | 10 |
| Infection | 10 |
| Contusion | 1 |

# TUS; thoracic ultrasound

**Table S4: Discrepancies**

| **Total number of discrepancies=96** | **Chest X-ray/CT** | **TUS^#^** |
| --- | --- | --- |
| 67 | Pleural effusion (request for paracentesis/drainage) | No or small pleural effusion |
| 6 | No pleural effusion | Pleural effusion |
| 7 | Pathology (eg infiltrate, nodule, mass) | Pathology not depicted |
| 16 | Pathology not depicted | New pathology (e.g consolidation,pneumothorax, abscess) |

# TUS; thoracic ultrasound

| Patients | 15 |
| --- | --- |
| ΤUS^#^ examinations | 42 |
| **Context of pregnancy** |  |
| Pregnancy | 26 |
| Post- partum | 7 |
| Ovarian hyperstimulation syndrome | 9 |
| **Request** |  |
| Q1: symptoms (fever, chest pain, dyspnea etc) (n) | 5 |
| Q2: pleural effusion evaluation and treatment (n) | 29 |
| Q3: follow up of a known disease/monitoring (n) | 8 |
| **Findings** |  |
| Normal (n) | 4 |
| Pleural effusion (n) | 33 |
| Pneumonia (n) | 5 |
| Consolidation(n) | 13 |
| Pulmonary embolism (n) | 1 |
| Diaphragm dysfunction (n) | 3 |
| Interstitial syndrome (n) | 1 |
| **Decision** |  |
| Normal (n) | 4 |
| Monitoring(n) | 26 |
| Paracentesis(n) | 10 |
| Surgery(n) | 2 |

**Table S5: ΤUS results related to pregnancy**

# TUS; thoracic ultrasound

Values are expressed as absolute numbers

**Table S6. TUS results related to malignancy**

| **Patients** |  | 376 |
| --- | --- | --- |
| **TUS^#^ examinations** |  | 433 |
| **Question of referral** |  |  |
|  | Symptoms and signs | 252 |
|  | Evaluation of pleural effusion | 136 |
|  | Pathologic Chest X-ray | 24 |
|  | Monitoring | 21 |
| **Echo findings** |  |  |
|  | Pleural effusion | 293 |
|  | Atelectasis  (Compressive or obstructive) | 233 |
|  | Paradoxical movement of the diaphragm | 168 |
|  | Diaphragm paralysis | 7 |
|  | Loculated effusion - empyema | 56 |
|  | Pneumonia | 10 |
|  | Cancer infiltrations | 10 |
|  | Haemothorax/Chylothorax/Pneumothorax | 7 |
| **Invasive Interventions** |  |  |
|  | Pleural effusion drainage | 186 |
|  | Diagnostic thoracentesis | 111 |
|  | Chest tube/Flexima placement-removal | 39 |
| **Noninvasive interventions** |  |  |
|  | Surgery suggestion-diuretics | 6 |
|  | Diagnostic (discrepancies) | 55 |
|  | Reevaluation-Monitoring | 25 |

# TUS; thoracic ultrasound

Values are expressed as absolute numbers

Figures Legends

Figure S1 a,b,c. Department of referral, question for TUS referral and thoracic ultrasound findings respectively

Figure S2a, b a) **Pneumothorax** in a patient presented with hypoxemia after many attempts of diagnostic thoracentesis at the ward. Red arrow: Pleural line. Blue arrows: A lines. M –mode displays the stratosphere-barcode sign. Yellow arrow. b) **Echo-guided drainage of a large pleural effusion**. The blue arrow indicate the needle linked with the acoustic shadow inside the pleural cavity. Notice the consolidated lung, the large pleural effusion (PE) and the diaphragm with well–defined borders and normal shape (white arrow).

Figures S3 a, b. a) **TUS after talc pleurodesis**. Multiple diaphragms and pockets in the pleural space. Notice the normal shape of the diaphragm indicating the absence of inflammatory process. b) **Left: B –mode clearly shows the three diaphragmatic layers**. Black arrow indicates the inner diameter corresponding to the diaphragmatic thickness. Right: Points of estimation of diaphragmatic thickness during the respiratory cycle. Red arrow: maximal thickness during inspiration and white arrow during expiration.

1a

1b

1c
