# Supplementary figures and images for: Thoracic ultrasound use in hospitalized and ambulatory adult patients: a quantitative picture
Source: Ultrasound J. 2024 Feb 21;16:11. doi: 10.1186/s13089-024-00359-4 (PMC10881936; doi:10.1186/s13089-024-00359-4)

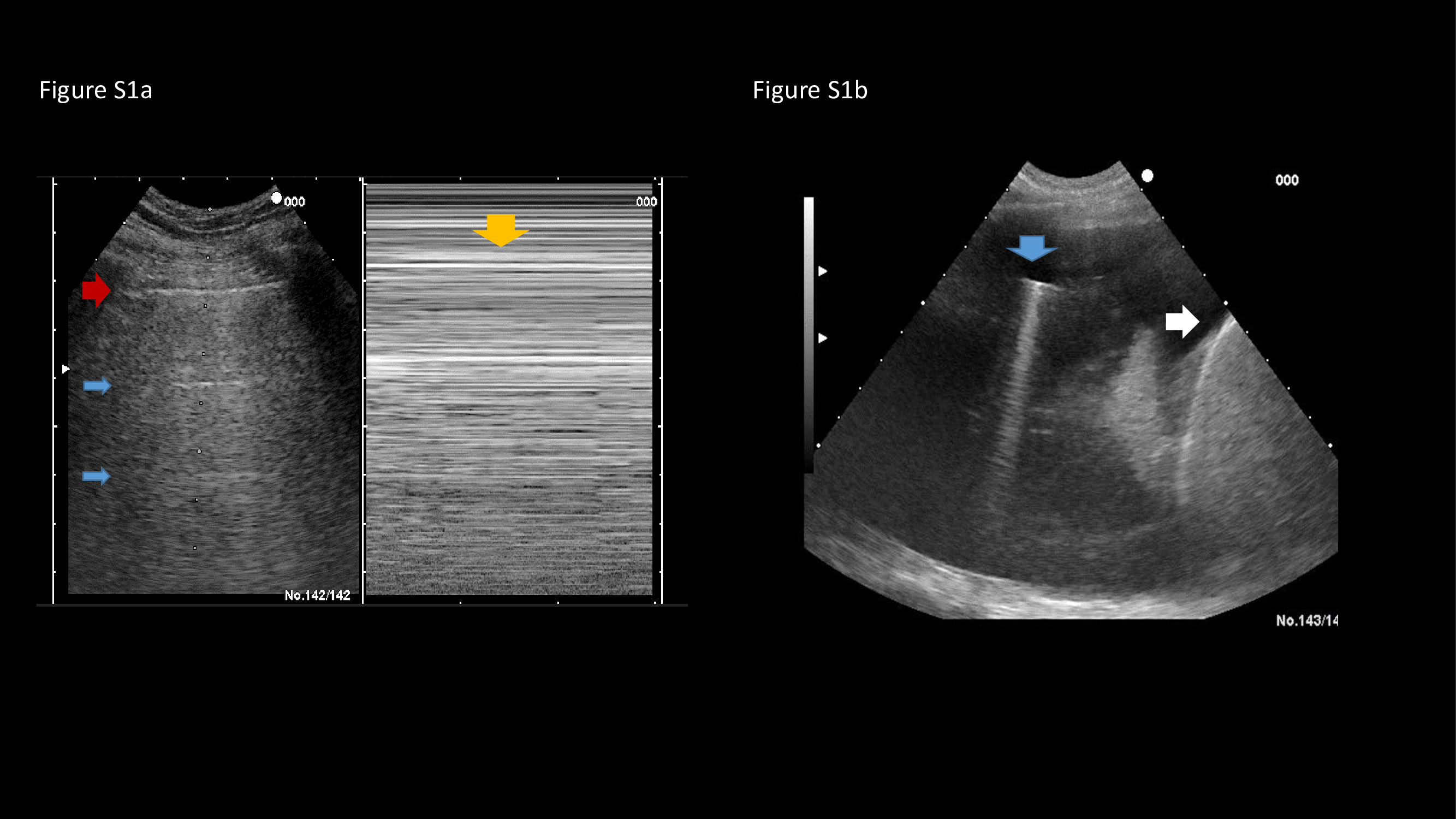

Supplement: Supplementary file 2 — Additional file 2: Figure S2. a Pneumothorax in a patient presented with hypoxemia after many attempts of diagnostic thoracentesis at the ward. Red arrow: Pleural line. Blue arrows: A lines. M-mode displays the stratosphere–barcode sign. Yellow arrow. b Echo-guided drainage of a large pleural effusion. The blue arrow indicate the needle linked with the acoustic shadow inside the pleural cavity. Notice the consolidated lung, the large pleural effusion (PE) and the diaphragm with well-defined borders and normal shape (white arrow). [file 13089_2024_359_MOESM2_ESM.png]

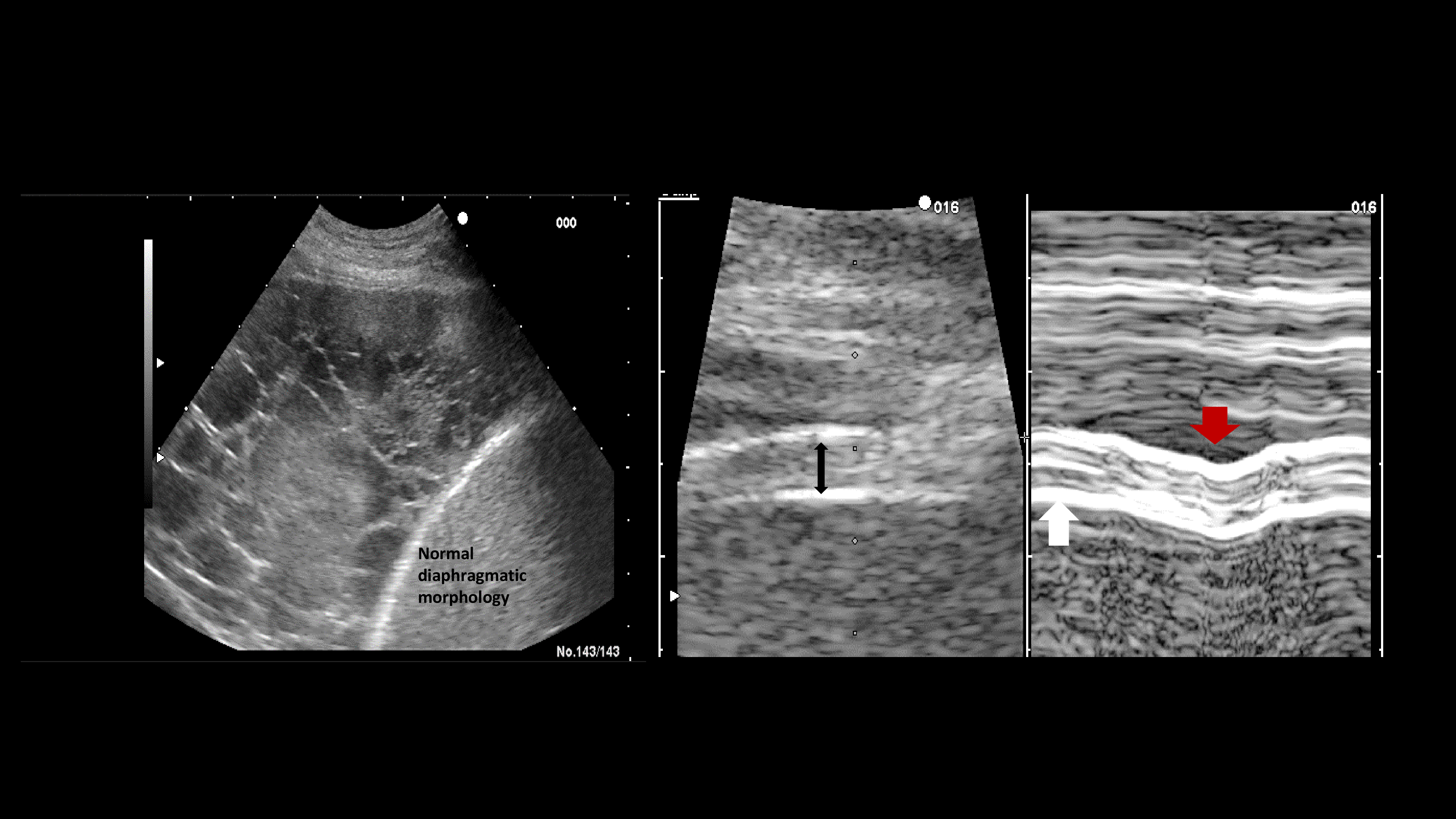

Supplement: Supplementary file 3 — Additional file 3: Figures S3. a TUS after talc pleurodesis. Multiple diaphragms and pockets in the pleural space. Notice the normal shape of the diaphragm indicating the absence of inflammatory process. b Left: B-mode clearly shows the three diaphragmatic layers. Black arrow indicates the inner diameter corresponding to the diaphragmatic thickness. Right: Points of estimation of diaphragmatic thickness during the respiratory cycle. Red arrow: maximal thickness during inspiration and white arrow during expiration. [file 13089_2024_359_MOESM3_ESM.png]
